# 552 Predictions Under Pressure: The Value of Intra-abdominal Hypertension in Forecasting Associated Complications During Fluid Resuscitation

**DOI:** 10.1093/jbcr/irad045.149

**Published:** 2023-05-15

**Authors:** Stacey Richerbach, Karen Richey, Kevin Foster

**Affiliations:** Arizona Burn Center at Valleywise Health, Phoenix, Arizona; Arizona Burn Center Valleywise Health, Phoenix, Arizona; AZ Burn Center at Valleywise Health, Phoenix, Arizona; Arizona Burn Center at Valleywise Health, Phoenix, Arizona; Arizona Burn Center Valleywise Health, Phoenix, Arizona; AZ Burn Center at Valleywise Health, Phoenix, Arizona; Arizona Burn Center at Valleywise Health, Phoenix, Arizona; Arizona Burn Center Valleywise Health, Phoenix, Arizona; AZ Burn Center at Valleywise Health, Phoenix, Arizona

## Abstract

**Introduction:**

The link between burn fluid resuscitation and intra-abdominal hypertension (IAH) is long-standing. Clinicians refer to measures of intra-abdominal pressure (IAP) to guide care of patients requiring resuscitation. In the non-burned critically ill patients mean IAP is 5-7 mmHg, yet more than half of patients with large burns have IAH with a pressure > 12mmHg. The purpose of this study was to determine the predictive value of IAH during resuscitation and to elucidate the prevalence of associated complications.

**Methods:**

This was a retrospective chart review of patients requiring fluid resuscitation over a five-year period. Patient stratification was based upon IAP, < 12 mmHg (NIAH) vs > 12 mmHg (IAH). Basic descriptive statistics were calculated.

**Results:**

Charts were reviewed for 138 patients with a total body surface area (TBSA) >20%. There were no significant differences for age, ethnicity, race, gender, nor mechanism of injury. Mean TBSA was greater for those with IAH 44.7% vs NIAH 29.6% (p < 0.0001). Diagnosis of inhalation injury was greater with IAH 37% vs NIAH 10% (p = 0.0009). Admission weight was greater in IAH 90.59 kg vs NIAH 80.34 kg (p=0.0169). Patients who arrive at our burn center sooner (IAH 105, NIAH 160 minutes; p = 0.033), were less likely to receive pre-hospital fluids (IAH 68%, NIAH 84%; p = 0.03850), and time from injury to the start of in-hospital resuscitation was lower (IAH 177 min, NIAH 245 min p = 0.02840). The average resuscitation duration was greater for IAH than for NIAH (34.4 h vs 28.04 h; p = 0.0008), as was total volume given (p < 0.0001), but resuscitation volume per kg/TBSA/hour was not statistically different (IAH 6.72 mL, NIAH 6.15 mL; p = 0.321). Mean urine output per hour between the groups was not significant (p 0.7699). Patients with IAH had more hours in the optimal range for urine output (6.6 h) than NIAH (4.95), (p = 0.041). No significant difference was revealed between the groups for eventual diagnosis of abdominal compartment syndrome (p = 0.06); compartment syndrome (p = 0.8346, ARDS (p = 0.242); laparotomy (p = 0.222), open abdomen (p = 0.060). Administration of vasopressors between the groups was insignificant (IAH 15%, NIAH 7%; p = 0.186), as was the use of CRRT (p = 0.787). Temperatures for hypothermic patients with IAH were significantly lower (34.5 c) than NIAH (35.1 c), (p = 0.0186) and patients with IAH were more likely to have an escharotomy than those in the NIAH group (IAH 43%, NIAH 16%; p = 0.0014).

**Conclusions:**

Most associated complications of fluid resuscitation were not more likely to occur with IAH as compared to NIAH. However, patients with IAH were more likely to warrant an escharotomy involving an extremity and had a lower temperature when hypothermic than the NIAH group.

**Applicability of Research to Practice:**

Further research is needed to evaluate the relationship between measures of IAP and associated complications.

